# Evidence-based organization and patient safety strategies in European hospitals

**DOI:** 10.1093/intqhc/mzu016

**Published:** 2014-02-26

**Authors:** Rosa Sunol, Cordula Wagner, Onyebuchi A. Arah, Charles D. Shaw, Solvejg Kristensen, Caroline A. Thompson, Maral Dersarkissian, Paul D. Bartels, Holger Pfaff, Mariona Secanell, Nuria Mora, Frantisek Vlcek, Halina Kutaj-Wasikowska, Basia Kutryba, Philippe Michel, Oliver Groene, on behalf of the DUQuE Project Consortium, N. Klazinga, D.S. Kringos, M.J.M.H. Lombarts, T. Plochg, M.A. Lopez, P. Vallejo, F. Saillour-Glenisson, M. Car, S. Jones, E. Klaus, S. Bottaro, P. Garel, M. Saluvan, C. Bruneau, A. Depaigne-Loth, A. Hammer, O. Ommen, H. Pfaff, D. Botje, A. Escoval, A. Lívio, M. Eiras, M. Franca, I. Leite, F. Almeman, H. Kus, K. Ozturk, R. Mannion, A. Wang, A. Thompson

**Affiliations:** 1 Avedis Donabedian Research Institute (FAD), Universitat Autonoma de Barcelona, Spain; 2 Red de investigación en servicios de salud en enfermedades crónicas REDISSEC, Spain; 3 NIVEL Netherlands Institute for Health Services Research, Utrecht, The Netherlands; 4 Department of Public and Occupational Health, EMGO+ Institute for Health and Care Research, VU University Medical Center, Amsterdam, The Netherlands; 5 Department of Epidemiology, Fielding School of Public Health, University of California, Los Angeles (UCLA), Los Angeles, CA, USA; 6 University of New South Wales, Australia; 7 Central Denmark Region & Center for Healthcare Improvements, Aalborg University, Denmark; 8 Palo Alto Medical Foundation Research Institute, Palo Alto, CA, USA; 9 Central Denmark Region & The Department of Clinical Medicine, Aalborg University, Denmark; 10 Institute of Medical Sociology, Health Services Research and Rehabilitation Science, University of Cologne, Germany; 11 Czech Accreditation Committee, Czech Republic; 12 Polish Society for Quality Promotion in Health Care, Poland; 13 Quality and Safety Department, Lyon University, Hospital Network, France; 14 London School of Hygiene and Tropical Medicine, UK

**Keywords:** patient safety, quality improvement, quality management, practice variations, appropriate healthcare, hospital care, effectiveness

## Abstract

**Objective:**

To explore how European hospitals have implemented patient safety strategies (PSS) and evidence-based organization of care pathway (EBOP) recommendations and examine the extent to which implementation varies between countries and hospitals.

**Design:**

Mixed-method multilevel cross-sectional design in seven countries as part of the European Union-funded project ‘Deepening our Understanding of Quality improvement in Europe’ (DUQuE).

**Setting and participants:**

Seventy-four acute care hospitals with 292 departments managing acute myocardial infarction (AMI), hip fracture, stroke, and obstetric deliveries.

**Main outcome measure:**

Five multi-item composite measures—one generic measure for PSS and four pathway-specific measures for EBOP.

**Results:**

Potassium chloride had only been removed from general medication stocks in 9.4–30.5% of different pathways wards and patients were adequately identified with wristband in 43.0–59.7%. Although 86.3% of areas treating AMI patients had immediate access to a specialist physician, only 56.0% had arrangements for patients to receive thrombolysis within 30 min of arrival at the hospital. A substantial amount of the total variance observed was due to between-hospital differences in the same country for PSS (65.9%). In EBOP, between-country differences play also an important role (10.1% in AMI to 57.1% in hip fracture).

**Conclusions:**

There were substantial gaps between evidence and practice of PSS and EBOP in a sample of European hospitals and variations due to country differences are more important in EBOP than in PSS, but less important than within-country variations. Agencies supporting the implementation of PSS and EBOP should closely re-examine the effectiveness of their current strategies.

## Introduction

Evidence-based medicine and the implementation of patient safety recommendations are considered two of the cornerstones for improving clinical care outcomes [1]. Agencies such as the National Institute for Health Care and Excellence (NICE) and the Scottish Intercollegiate Guidelines Network (SIGN) in the UK and the French Haute Autorité de Santé (HAS) have long-standing clinical pathway guidelines including measures and descriptors for the delivery of high-quality care based on the implementation of evidence-based clinical recommendations and organizational practices [2–4], such as access to 24/7 diagnostic and intervention facilities and systems for triage and transfer to a specialist unit.

International patient safety efforts include the Global Patient Safety Alliance launched by the World Health Organization (WHO) and the Health Care Quality Indicator Project led by the Organization for Economic Co-operation and Development (OECD) [5]. In Europe, the Safety Improvement for Patients in Europe (SImPatIE) project established a common European vocabulary and set of indicators and internal and external instruments to improve safety in healthcare [6] and the European Network for Patient Safety (EUNetPaS) created an umbrella network of all European Union (EU) member states and stakeholders to enhance collaboration in the field of patient safety [7]. Currently, the joint action on Patient Safety and Quality of Care is identifying activities and tools for mutual learning among all EU member states [8].

In recent decades, clinical managers have sought to adopt the above recommendations and improve patient care through system design, guidelines and protocols, adverse event (AE) reporting, and risk management. There is, however, limited evidence that the efforts undertaken have led to significant improvements in compliance with evidence-based medicine or reduction in AEs [9–12]. Another concern is the scarcity of information on the implementation of patient safety strategies (PSS) and evidence-based organization of pathways (EBOP) in European hospitals. Compliance with these best practices in EU countries is also an important policy issue as the recent EU directive on cross-border healthcare reinforces the rights of citizens to seek care in another member state [13].

Our objectives are to describe the implementation of PSS and EBOP in a sample of European hospitals and to explore the extent to which individual countries, hospitals, and department level effects explain the variations observed.

## Methods

### Setting

This study was conducted as part of the Deepening our Understanding of Quality improvement in Europe (DUQuE) research project, whose conceptual framework and design is detailed elsewhere [14]. We performed a cross-sectional, multilevel study in which patient-level measurements are nested in hospital departments, which, in turn, are nested in hospitals in seven European countries (Czech Republic, France, Germany, Poland, Portugal, Spain, and Turkey). Thirty hospitals were randomly selected in each country and of these, 12 were selected, also randomly, for comprehensive data collection at department and patient level (maximum of 84 hospitals). Pathway and patient-level data were collected for four areas: acute myocardial infarction (AMI), obstetric deliveries, hip fracture, and stroke. Ethical approval was obtained from the Bioethics Committee of the Catalan Health Department (Spain) and each country complied with confidentiality issues according to national legislation or country-specific standards of practice.

### Measures used in the analysis

PSS were identified through a mapping process of patient safety recommendations drawn from the WHO High 5s project [15], the Joint Commission International Patient Safety Goals [16], Required Organizational Practices from Accreditation Canada [17], and guidelines from intergovernmental organizations such as the Council of Europe [18], the Council of the European Union [19], and the WHO (World Alliance on Patient Safety program) [20]. Selection of PSS was based on the frequency of mention in the reviewed documents and coverage of key safety areas (infection control, medication, life support, AE reporting systems, and security). Selection was also based on the underlying evidence that a patient safety practice prevents or reduces AEs. Eleven PSS items were analyzed. Nine were common to the four pathways (elimination of potassium chloride concentrate from ward stocks, use of wristband identification, needle disposal boxes, hand hygiene promotion/reminders, alcohol-based hand rubs, resuscitation flow charts, crash carts, AE reporting systems, use of AE reports in department quality of care evaluations) and two were specific to obstetric deliveries (neonate identification and secure nursery access). We decided not to analyze safe injection practices as this recommendation was considered to be generally implemented in the countries analyzed. EBOP items were derived from NICE quality standards and SIGN audit tools for the areas relevant to our study [21–24]. Although specific EBOP recommendations differed for the four areas, they followed a generic care pathway design and included items related to admission, acute care, rehabilitation (where appropriate), and discharge. The final measures included 5–10 pathway-specific items (Tables 3 and 4) [25]. We assumed that hospital characteristics (size, ownership, and teaching status) and extent of implementation of quality management (QM) systems at hospital level could influence the implementation of PSS and EBOP at department level. In the DUQuE project, hospital-level QM systems are assessed using three measures: the QM Systems Index (QMSI) (having QM systems in place), the QM Compliance Index (QMCI) (compliance with Plan-Do-Check/Study-Act cycle), and the Clinical Quality Implementation Index (CQII). The validation and results of these measures are reported elsewhere [26, 27].

### Data collection

Data on PSS, EBOP, QMSI, and CQII were gathered during a 1.5-day onsite audit with visits to all clinical areas, including emergency rooms, contemplated in this study. The audits were performed by external auditors (14 in total) with experience in quality and safety management and hospital accreditation and no relationship with the hospital in question. A centralized ‘teach the teacher’ session was carried out with leading auditors to unify the implementation of the external assessment tool. Training included theoretical and practical information, instructions on the main aspects to be assessed, and scoring guidance.

All items included in PSS and EBOP were assessed by direct observation and rated on a five-point-Likert scale ranging from ‘no or negligible compliance’ (score 0) to ‘full compliance’ (score 4). Explicit criteria were developed to rank each of these positions for each item. Data were collected between May 2011 and February 2012.

### Statistical analysis

We used univariate and bivariate statistics to report the implementation of PSS and EBOP. Descriptive tables show the number and percentage of departments rated as fully compliant (≥90%) for each item. In order to report the country range for each item, we calculated the percentage of hospitals in each country reported being fully compliant for each item. The country range shows the minimum and maximum of this value across the seven countries included in our analysis. We computed an overall score from the mean score for all items on each specific scale to be used in the multilevel regression models.

We used multivariable adjusted linear mixed models to decompose the outcome variance for PSS and EBOP at each pathway. The model for PSS included two random intercepts for country and hospitals nested within country to account for clustering of hospitals within countries and clustering of the four pathways within hospitals. This allowed us to estimate between-country, between-hospital, and within-hospital variances. The PSS model also included fixed effects for pathway, hospital characteristics (ownership, hospital size, and teaching status), and QM measures at hospital level (QMSI, QMCI, and CQII). A second set of models stratified by pathway were also used to estimate the contribution of between-country and within-country variances to the total variance of EBOP. These models included a random intercept by country to account for clustering of hospitals within countries and fixed effects for hospital characteristics (ownership, hospital size, and teaching status) and QM measures at hospital level (QMSI, QMCI, and CQII). These statistical analyses were carried out in SAS (version 9.3, SAS Institute Inc., NC, USA, 2001) [28].

## Results

Seventy-four hospitals from seven countries provided valid data. We analyzed 292 pathways. The hospital and pathway characteristics are shown in Table 1.

**Table 1:** Characteristics of hospitals by care pathway

	AMI (*n* = 72)	Obstetric deliveries (*n* = 72)	Hip fracture (*n* = 74)	Stroke (*n* = 74)
	*n* (%)	*n* (%)	*n* (%)	*n* (%)
Teaching hospital				
Yes	32 (44.4)	33 (45.8)	33 (44.5)	33 (44.5)
No	40 (55.5)	39 (54.1)	41 (55.4)	41 (55.4)
Ownership				
Public	59 (81.9)	58 (80.5)	59 (79.7)	59 (79.7)
Private or mixed	13 (18.0)	14 (19.4)	15 (20.2)	15 (20.2)
Number of beds				
<200	7 (9.7)	6 (8.3)	7 (9.4)	7 (9.4)
200–500	21 (29.1)	22 (30.5)	22 (29.7)	22 (29.7)
500–1000	30 (41.6)	31 (43.0)	31 (41.8)	31 (41.8)
>1000	14 (19.4)	13 (18.0)	14 (18.9)	14 (18.9)
Teaching department status				
Yes	39 (54.1)	42	45 (60.8)	40 (54.0)
No	33 (45.8)	30	29 (39.1)	34 (45.9)

AMI, acute myocardial infarction.

Table 2 shows compliance with PSS by department together with the average country range for each item. Over 90% of patients were identified by wristband in 43.0% of AMI wards (31/72), compared with 59.7% of maternity wards (43/72). No significant differences between-department were observed for compliance with patient identification recommendations, but the differences between country averages were substantial, with figures ranging from 0 to 91.7%. Recommendations such as the use of needle disposal boxes and alcohol-based rubs were met in over 85% of all departments, and the variability between countries was lower (66.7–100.0%). Over two-thirds of maternity wards still had potassium chloride concentrate in their medication stocks, and the figures were even higher in the other departments. AE reporting systems were available in just one-third of the departments audited and reports were used for quality of care evaluations in just half of these.

**Table 2:** Compliance with PSS by department and country ranges^a^

	AMI (*n* = 72)	Country range	Obstetric deliveries (*n* = 72)	Country range	Hip (*n* = 74)	Country range	Stroke (*n* = 74)	Average country range	*P*-value^b^
	*n* (%)	%	*n* (%)		*n* (%)	%	*n* (%)	%	
Patient wristbands	31 (43.0)	25.0–63.6	43 (59.7)	0.0–90.9	37 (50.0)	25.0–75.0	41 (55.4)	25.0–91.7	0.2131
Needle disposal boxes	65 (90.2)	75.0–100.0	67 (93.0)	75.0–100.0	66 (89.1)	66.7–100.0	65 (87.8)	66.7–100.0	0.7537
Hand hygiene promotion/reminder	55 (76.3)	54.6–100.0	53 (73.6)	54.6–100.0	53 (71.6)	58.3–83.3	50 (67.5)	50.0–91.7	0.6809
Alcohol-based hand rubs	65 (90.2)	75.0–100.0	65 (90.2)	66.7–100	66 (89.1)	75.0–100.0	68 (91.8)	75.0–100.0	0.9567
No potassium chloride concentrate in patient services areas	11 (15.2)	0.0–50.0	22 (30.5)	8.3–50.0	7 (9.4)	0.0–41.7	8 (10.8)	0.0–41.7	0.0020
Resuscitation flow charts	14 (19.4)	0.0–100.0	11 (15.2)	0.0–75.0	10 (13.5)	0.0–75.0	10 (13.5)	0.0–50.0	0.7272
Crash cart	40 (55.5)	0.0–90.9	37 (51.3)	8.3–83.3	34 (45.9)	0.0–75.0	37 (50.0)	0.0–81.8	0.7111
Adverse event reporting system	25 (34.7)	8.3–75.0	23 (31.9)	16.7–75.0	22 (29.7)	0.0–54.6	22 (29.7)	0.0–75.0	0.9037
Adverse event reports used for quality of care evaluations	11 (15.2)	0.0–25.0	20 (27.7)	8.3–75.0	9 (12.1)	8.3–50	12 (16.2)	0.0–33.3	0.0737
Overall score, mean (SD)	2.6 (0.5)		2.7 (0.6)		2.5 (0.5)		2.5 (0.6)		

^a^Compliance is shown by number (%) of departments reporting full compliance; country ranges show minimum–maximum compliance rates (%) based on country averages.

^b^*P*-value for differences in items across pathways (*χ*^2^ test).

Tables 3 and 4 show results for EBOP. Although a high proportion of areas treating AMI patients (83.6%; country range, 66.7–100%) have immediate, around-the-clock access to a specialist physician to assess the need for coronary revascularization, only 56.0% (country range, 18.2–85.7%) have arrangements for patients to receive thrombolysis within 30 min of arrival at the hospital.

**Table 3:** Compliance with evidence-based organization of care pathway recommendations for AMI and stroke^a^

Item	*n* (%), full compliance	Average country range^a^ (%)
Acute myocardial infarction departments (*n* = 66)		
1. There are written criteria and procedures for fast-track admission and treatment of patients presenting with acute chest pain	36 (54.5)	18.2–90.9
2. Arrangements ensure that eligible STEMI (S-T elevation Myocardial Infarction) patients can receive thrombolysis within 30 min of arrival at the hospital	37 (56.0)	18.2–85.7
3. Immediate access is available 24/7 to a specialist physician to determine whether coronary revascularization is appropriate	57 (86.3)	66.7–100.0
4. Facilities are immediately available for performance of and transport for emergency coronary angiography	48 (72.7)	40.0–90.9
5. Facilities are immediately available for performance of and transport for percutaneous coronary intervention	44 (66.6)	36.4–81.8
Overall score, mean (SD)	3.2 (0.9)	
Stroke departments (*n* = 74)		
1. There is an agreed procedure for appropriate patients to be directly transported by ambulance personnel to a stroke unit	42 (56.7)	36.4–100.0
2. Agreed procedures ensure that patients with suspected stroke are assessed for receiving thrombolysis, if clinically indicated	55 (74.3)	41.7–100.0
3. A thrombolysis service is available 7 days a week in the hospital or by formal arrangement elsewhere	62 (83.7)	58.3–100.0
4. Agreed procedures ensure that patients with acute stroke have their swallowing screened by a specially trained healthcare professional	35 (47.2)	0.0–100.0
5. Protocols and procedures are available for patients to receive brain imaging within 1 h of arrival at the hospital	46 (62.1)	25.0–91.7
6. Protocols are in place to ensure documented multidisciplinary goals are agreed within 5 days of admission to hospital	31 (41.8)	8.3–66.7
7. There is immediate access (1 h) to a specialist acute stroke unit (or area) for those with persisting neurological symptoms	51 (68.9)	50.0–83.3
Overall score, mean (SD)	3.0 (1.0)	

^a^Minimum–maximum percentage of fully compliant hospitals by country used to determine country ranges.

**Table 4:** Compliance with evidence-based organization of care pathway recommendations for obstetric deliveries and hip fracture^a^

Item	*n* (%), full compliance	Average country range (%)
Obstetric deliveries (*n* = 72)		
1.A structured, accurate record of all events during the antenatal childbirth and postnatal periods is maintained for every woman and child	66 (91.6)	58.3–100.0
2. All women who have epidural analgesia or an operative delivery have their pain assessed using a pain assessment tool approved by the hospital	42 (58.3)	8.3–100.0
3. There is prompt access to ultrasound facilities with trained staff	72 (100)	100.0–100.0
4. There is a procedure that guarantees that all women who are identified in the screening program as at risk of rhesus disease are properly managed	44 (61.1)	25.0–100.0
5. Each woman receives one-to-one midwifery care from a trained midwife during established labor and childbirth	63 (87.5)	0.0–100.0
6. Epidural analgesia is available at all times	61 (84.7)	58.3–100.0
7. Adult intensive care facilities and specialist medical back-up are available onsite	70 (97.2)	90.9–100.0
8. Patient monitoring equipment and clinical expertise in its management are available within the obstetric unit	71 (98.6)	91.7–100.0
9. There is a system in place to ensure that anesthetic and theater services respond within 30 min to obstetric emergencies and expedite delivery in the event of maternal or fetal compromise	69 (95.8)	83.3–100.0
10. All babies are clinically examined prior to discharge from hospital and/or within 72 h of birth by a suitably qualified healthcare professional	71 (98.6)	90.9–100.0
Overall score, mean (SD)	3.7 (0.3)	
Hip fracture (*n* = 74)		
1. The guidelines require that medical staff assess patients suspected of having a fractured hip within 1 h of arrival in the emergency department or of the incident if the patient was already in hospital	27 (36.4)	0.0–75.0
2. The guidelines require a multidisciplinary assessment plan and individual goals for rehabilitation to be documented within 24 h postoperatively	16 (21.6)	0.0–75.0
3. Magnetic resonance imaging (MRI) is immediately available if hip fracture is suspected, despite negative plain X-rays	40 (54.0)	0.0–100.0
4. The guidelines require that all patients presenting with a fragility (pathological) fracture are managed on a ward with routine access to orthogeriatric medical support	14 (18.9)	0.0–75.0
5. Whenever clinically appropriate, surgery is performed within 48 h of admission	45 (60.8)	33.3–100.0
6. Guidelines require that all patients undergoing hip fracture surgery receive antibiotic prophylaxis	51 (68.9)	0.0–100.0
7. Guidelines require that, if the patient's overall medical condition allows, mobilization begins within 24 h postoperatively	28 (37.8)	0.0–75.0
Overall score, mean (SD)	2.3 (1.0)	

^a^Minimum–maximum percentage of fully compliant hospitals by country used to determine country range.

Findings for fast-track admission, timely intervention, access to specialist skills, and diagnostic and treatment facilities were similar for stroke and AMI patients. Screening of swallowing by a specially trained healthcare professional in acute stroke patients was limited (47.2%), with dramatic differences in compliance (0–100%) between countries.

Delivery departments showed the best compliance with EBOP, with prompt access to ultrasound facilities available in all 72 departments. In one country, all 10 criteria analyzed were met in all the hospitals, but in the other countries, fewer than half of the departments complied with recommendations regarding pain assessment, one-to-one midwifery care, and screening for Rhesus disease.

Hip fracture departments showed the lowest overall compliance with EBOP and also the highest between-country variability. Six of the seven criteria assessed were not met in at least one country, but were fully met in others. Where indicated, 61% of departments (country range, 33.3–100%) perform hip surgery within 48 h of admission.

Table 5 shows estimates for the covariance parameters from the linear mixed models described above. In the case of PSS (Model 1 of Table 5), after adjusting for pathway and hospital-level predictors, country differences in our sample accounted for 0% of the total variance. A larger amount of the total variance (65.9%) was explained by differences between hospitals within countries.

**Table 5:** Country- and hospital-level variances (as percentages of the total variance) of PSS and evidence-based organization of the care pathway (EBOP) scores

	Between-country variability	Within-country (between-hospital) variability	Within-hospital (between-department) variability	Total variance
	Variance (%)	Variance (%)	Variance (%)	
^a^Model 1. PSS modeled as a function of pathway, hospital-level quality implementation, ownership status, teaching status, and size of hospitals with random intercepts for country and hospital nested within country	0 (0)	0.1827 (65.9)	0.0943 (34.1)	0.2770
^b^Model 2. AMI-EBOP modeled as a function of hospital-level quality implementation, ownership status, teaching status, and size of hospitals with random intercepts for country and hospital nested within country	0.0685 (10.1)	0.6127 (89.9)	N.A.	0.6812
^b^Model 3. STROKE-EBOP modeled as a function of hospital-level quality implementation, ownership status, teaching status, and size of hospitals with random intercepts for country and hospital nested within country	0.3078 (31.8)	0.6603 (68.2)	N.A.	0.9681
^b^Model 4.OBSTETRIC DELIVERIES-EBOP modeled as a function of hospital level quality implementation, ownership status, teaching status, and size of hospitals with random intercepts for country and hospital nested within country	0.0557 (40.0)	0.0835 (60.0)	N.A.	0.1392
^b^Model 5. HIP FRACTURE-EBOP modeled as a function of hospital-level quality implementation, ownership status, teaching status, and size of hospitals with random intercepts for country and hospital nested within country	0.7226 (56.3)	0.5611 (43.7)	N.A.	1.2837

^a^Linear mixed regression with random intercept by country and hospital nested within country, and fixed effects for pathway, hospital-level quality implementation (QMSI, QMSCI, CQII), and hospital characteristics (number of beds, hospital ownership, hospital teaching status).

^b^Linear mixed regression by pathway with random intercept by country, and fixed effects for hospital-level quality implementation (QMSI, QMSCI, CQII) and hospital characteristics (number of beds, hospital ownership, hospital teaching status).

Models 2–5 in Table 5 show variance decomposition results for EBOP. For AMI, obstetric deliveries, and stroke, the proportion of variance explained by between-country variation was considerable, but less (10.1% in AMI, 40.0% in obstetric deliveries, and 31.8% in stroke) than that explained by differences within countries. Between-country differences explained a greater proportion of the variance of EBOP in the hip fracture pathway (56.3%) compared with the other pathways.

## Discussion

We assessed the implementation of PSS and EBOP and possible sources of variation in a large sample of European hospitals. Our results indicate that neither PSS nor EBOP recommendations are routinely followed and that there are substantial differences between departments and hospitals. Our findings raise serious concerns regarding the delivery of optimal care and indicate substantial room for improvement. Unfortunately, there is a scarcity of both literature on compliance with PSS and EBOP recommendations in Europe and comparable indicators. The criteria we used in our assessment were based on guidelines from international expert groups and on research findings and empirical evidence, which in many cases, date back several years.

Our results show that compliance with certain key recommendations is far from ideal. Just one in three wards (in some countries, 1 in 10) have removed potassium chloride concentrate solution from their general stocks, even though this practice was recommended by the UK National Patient Safety Agency as early as 2002 [29] and has since been advocated by many other safety agencies. Other areas, in contrast, seem to have improved. In a similar external ward evaluation conducted in 2007 in eight EU countries, the percentage of wristband-identified patients in medical and surgical wards was 22–30% [30]. In our study, 43.0–59.7% of wards have over 90% of patients adequately identified. While our figures indicate an improvement, they are still far below the compliance rates that would be expected for such a basic and crucial safety recommendation [31].

Prompt, timely intervention is a key component of the care pathway in most conditions. In our study, only 56.0% of clinical areas treating AMI patients (country range, 18.2–85.7%) had verifiable arrangements for patients to receive thrombolysis within the recommended timeframe. While some EBOP recommendations have been fully implemented in some departments and hospitals, in others, they are scarcely visible. The variations in the adoption of these recommendations are similar to those reported for the uptake of research evidence [32] and guidelines in clinical practice [33]. With the exception of timely access to specialized diagnostic and treatment facilities, most of the gaps identified in our study could be remedied with minimum investment and simple strategies such as wristband identification, removal of potassium chloride stocks, or the implementation of swallowing screening protocols for stroke patients. Barriers to implementation might include conflicting messages from different sources and a lack of information in local languages. Additionally, poor compliance with PSS and EBOP recommendations might be influenced by the fact that they are not a legal or contractual requirement and also perhaps by the fact that agencies advocating their implementation have overly focused on the passive dissemination of knowledge, and ignored contextual factors that might facilitate or hinder implementation [34].

In the area of patient safety, differences between hospitals within a country accounted for a greater proportion of the total variance (65.9%), while between-country differences accounted for 0% of the total variance after adjusting for hospital-level predictors. Country-level compliance could be similar, because of a possible direct effect on hospitals of patient safety recommendations by international agencies, and low influence by country policies. In the case of EBOP, between-country differences play more important role and explained at least 30% of the total variance for three of four pathways. One possible explanation could be that EBOP is linked to consensus statements issued by specialist groups that are disseminated nationally through scientific meetings, country-wide recommendations, etc. Variations between countries were lower in areas where more level A evidence was available at the time of the study (e.g. AMI).

Our study has some limitations. Our findings cannot be generalized to the EU as a whole. Although random sampling was performed in each country, the conclusions are not generalizable because of our study design, the limited sample sizes and substantial differences in the percentage of refusals from one country to the next. It was not the aim of this study to present findings that could be generalized or compared at the country level, although such information would logically be of interest to policymakers. Rather, given that all EU hospitals potentially deliver services to all European citizens in the context of cross-border care, we decided to explore potential sources of variation in the measures. Here, again, sample size limitation could be important in the findings analysis because it could contribute to unstable variance estimates. It should also be noted that the instruments used to evaluate PSS and EBOP are new and need further testing and refinement before their widespread use can be recommended. We also did not analyze inter-rater reliability, but potential bias from this source was limited by organizing centralized training sessions and using a limited number of auditors per country. Despite the limitations of our study, we performed one of the largest ever European studies and used an identical set of indicators to measure PSS and EBOP across seven countries.

Regarding implications for policy, healthcare providers, purchasers, and insurers should focus not only on bundles of care but also on timing, as prompt delivery of care can optimize both costs and benefits. Delays can be reduced through investment in skills and equipment, adoption of triage and fast-track strategies, and early transfer of patients to specialist centers where indicated. In stroke patients, for instance, early differentiation between ischemic and haemorrhagic causes is critical to early intervention and faster recovery. In our study, the structures required to grant this ‘window of opportunity’ were in place in just 62.1% of departments, and in some countries, this percentage was as low as 25%. Findings from the Stroke Improvement National Audit Programme of the Royal College of Physicians of London show that the percentage of stroke patients receiving a brain scan within 24 h of admission increased from 42% in 2006 to 90% in 2012 [35], demonstrating that changes in hospital practices can clearly benefit patients. Making PSS and EBOP recommendations, department requirements could help to improve the situation in many areas.

The EU directive on cross-border healthcare [13] requires member states of treatment (where cross-border healthcare is provided) to develop quality and safety standards and guidelines but allows member states of affiliation (where patients are insured) to refuse authorization for treatment in another country for a number of reasons, including exposure ‘with reasonable certainty to a patient-safety risk that cannot be regarded as acceptable’. There is no definition of acceptable risk, but evidence of failure to adopt well-evidenced guidelines on systems to protect patients may offer legitimate grounds for denial of authorization. In our study, 224 (83%) of the 292 departments would be considered ‘unsafe’ if removal of potassium chloride concentrate from general ward stocks was considered essential, and in the case of wristband identification, 140 departments (48%) would not pass the test.

The fact that country differences seem to play a minor role in explaining the variations observed in the implementation of PSS has important policy consequences for EU member states because, at least based on our findings, there are no indications that citizens have a better chance of receiving safer care outside their country. In the case for EBOP, country differences account for more than one-third of variation in three conditions, but within-country differences were the main source of variation, except for hip fracture. This has also important consequences when patients seek for care outside of their country. These findings highlight the importance of compiling public data on the extent of implementation of PSS and EBOP to help citizens to choose hospitals not only outside their country of origin but also within their own country.

## Conclusions

This study shows significant gaps between knowledge and practice of patient safety and evidence-based organization in a sample of European hospitals. Our findings have implications for both policy and individual welfare as they suggest that a substantial proportion of European citizens are at risk of receiving suboptimal care and compliance with PSS and EBOP varied considerably from one hospital to the next in the same country and less between countries. Citizens need to be informed of these variations and to understand the importance of having access to information on hospital and department compliance with PSS and EBOP recommendations both inside and outside their countries. Direct comparisons were not feasible in our study because of the small sample sizes, but future research could focus on comparative analysis and explore in greater depth the causes of variability within regions, states, and even across borders.

## Funding

This study was funded by the European Commission's Seventh Framework Programme FP7/2007–2013 under grant agreement n° 24188. Funding to pay the Open Access publication charges for this article was provided by European Commission's Seventh Framework Programme FP7/2007–2013 under grant agreement n° 24188.

## Appendix

The DUQuE Project Consortium comprises: N. Klazinga, D.S. Kringos, M.J.M.H. Lombarts, and T. Plochg (Academic Medical Centre—AMC, University of Amsterdam, The Netherlands); M.A. Lopez, M.S., R.S., and P. Vallejo (Avedis Donabedian University Institute-Universitat Autónoma de Barcelona FAD, Red de investigación en servicios de salud en enfermedades crónicas REDISSEC, Spain); P.D.B. and S.K. (Central Denmark Region and Center for Healthcare Improvements, Aalborg University, Denmark); P.M. and F. Saillour-Glenisson (Comité de la Coordination de l'Evaluation Clinique et de la Qualité en Aquitaine, France); F.V. (Czech Accreditation Committee, Czech Republic); M. Car, S. Jones, and E. Klaus (Dr Foster Intelligence—DFI, UK); S. Bottaro and P. Garel (European Hospital and Healthcare Federation-HOPE, Belgium); M. Saluvan (Hacettepe University, Turkey); C. Bruneau and A. Depaigne-Loth (Haute Autorité de la Santé-HAS, France); C.D.S. (University of New South Wales, Australia); A. Hammer, O. Ommen, and H. Pfaff (Institute of Medical Sociology, Health Services Research and Rehabilitation Science, University of Cologne-IMVR, Germany); O.G. (London School of Hygiene and Tropical Medicine, UK); D. Botje and C.W. (The Netherlands Institute for Health Services Research—NIVEL, The Netherlands); H.K.-W. and B.K. (Polish Society for Quality Promotion in Health Care-TPJ, Poland); A. Escoval and A. Lívio (Portuguese Association for Hospital Development-APDH, Portugal) and M. Eiras, M. Franca, and I. Leite (Portuguese Society for Quality in Health Care-SPQS, Portugal); F. Almeman, H. Kus, and K. Ozturk (Turkish Society for Quality Improvement in Healthcare-SKID, Turkey); R. Mannion (University of Birmingham, UK); O.A.A., M.D., C.A.T., and A. Wang (University of California, Los Angeles—UCLA, USA); A. Thompson (University of Edinburgh, UK).
